# Immunological Pathways Triggered by *Porphyromonas gingivalis* and *Fusobacterium nucleatum*: Therapeutic Possibilities?

**DOI:** 10.1155/2019/7241312

**Published:** 2019-06-24

**Authors:** Kívia Queiroz de Andrade, Cássio Luiz Coutinho Almeida-da-Silva, Robson Coutinho-Silva

**Affiliations:** ^1^Immunobiology Program, Institute of Biophysics Carlos Chagas Filho, Federal University of Rio de Janeiro, Rio de Janeiro, RJ 21941-902, Brazil; ^2^Department of Biomedical Sciences, University of the Pacific, Arthur A. Dugoni School of Dentistry, San Francisco, CA 94103, USA

## Abstract

*Porphyromonas gingivalis* (*P. gingivalis*) and *Fusobacterium nucleatum* (*F. nucleatum*) are Gram-negative anaerobic bacteria possessing several virulence factors that make them potential pathogens associated with periodontal disease. Periodontal diseases are chronic inflammatory diseases of the oral cavity, including gingivitis and periodontitis. Periodontitis can lead to tooth loss and is considered one of the most prevalent diseases worldwide. *P. gingivalis* and *F. nucleatum* possess virulence factors that allow them to survive in hostile environments by selectively modulating the host's immune-inflammatory response, thereby creating major challenges to host cell survival. Studies have demonstrated that bacterial infection and the host immune responses are involved in the induction of periodontitis. The NLRP3 inflammasome and its effector molecules (IL-1*β* and caspase-1) play roles in the development of periodontitis. We and others have reported that the purinergic P2X7 receptor plays a role in the modulation of periodontal disease and intracellular pathogen control. Caspase-4/5 (in humans) and caspase-11 (in mice) are important effectors for combating bacterial pathogens via mediation of cell death and IL-1*β* release. The exact molecular events of the host's response to these bacteria are not fully understood. Here, we review innate and adaptive immune responses induced by *P. gingivalis* and *F. nucleatum* infections and discuss the possibility of manipulations of the immune response as therapeutic strategies. Given the global burden of periodontitis, it is important to develop therapeutic targets for the prophylaxis of periodontopathogen infections.

## 1. Introduction

Oral bacteria in dental biofilms contribute to the initiation and progression of periodontal diseases (PD) via exacerbated host inflammatory responses to these bacteria [1, 2]. PD are chronic inflammatory diseases of the periodontium (supporting structures around the teeth: gingiva, periodontal ligaments, and alveolar bone [3]), including gingivitis and periodontitis. Gingivitis is the initial reversible inflammatory lesion in the soft tissues surrounding the teeth, and periodontitis results from a combination of factors that leads to periodontium destruction, often causing irreversible bone resorption and tooth loss [3]. It affects nearly half of the United States population [4], and severe periodontitis is the 6^th^ most prevalent disease worldwide [5]. Periodontitis has a high impact on public health because of its long and expensive treatment. Furthermore, periodontitis is associated with several systemic diseases, including diabetes mellitus, cardiovascular diseases, and atherosclerosis, as we and others reviewed elsewhere [6, 7].

In healthy individuals, there is an established homeostasis between immunity and oral cavity microorganisms that do not cause diseases [8]. Oral epithelial and immune cells contribute directly and indirectly to maintain this equilibrium [9]. The loss of homeostasis due to dental plaque formation along with genetic, hormonal, and host behavioral factors make the individual susceptible to PD. Furthermore, the absence or decrease of an effective innate immune response by some cells stimulated by *P. gingivalis* LPS can greatly increase the proliferation of various bacterial species with the formation of biofilms at the root of the tooth, leading to exacerbated inflammation in the tissues [10].

*P. gingivalis*, a nonmotile, non-spore-forming Gram-negative bacterium, has the ability to induce dysbiosis in the oral microbiota (an imbalance among microbial species) [3, 11, 12]. *F. nucleatum* is one of the most common species in the human gingival sulcus; its prevalence increases with the severity of PD and the progression of inflammation [13, 14]. *F. nucleatum* serves as a true bridge, connecting initial and later bacterial colonizers, thereby favoring the formation of dental plaques [15]. When *F. nucleatum* is not present, the number of late colonizers is significantly lower [15].

Conventional clinical treatment for periodontitis consists initially of mechanical bacterial removal (scaling and root planning), thereby reducing the contact of bacterial agents with inflammatory and noninflammatory cells in the oral cavity. However, this procedure may not be sufficient to generate clinical improvement. In this context, several signaling pathways are involved in the progression of PD; therefore, therapies that modulate these pathways may help prevent the development of PD and consequently avoid bone loss [16].

The exact molecular host response events against *P. gingivalis* and *F. nucleatum* are not fully understood; nevertheless, understanding of these mechanisms is essential for the identification of therapeutic targets aiming to prevent and treat periodontitis. In this context, there are some immunological pathways that have been demonstrated to be involved in the development of periodontitis and in infections with periodontopathogens. In this respect, we and others previously demonstrated the role of the NLRP3 inflammasome in the development of periodontitis [17, 18]. Furthermore, it is known that purinergic signaling via the P2X7 receptor is one of the important pathways for the activation of the NLRP3 inflammasome and control of intracellular infections, including *P. gingivalis* infections [19, 20]. The activation of this inflammasome leads to caspase-1 maturation, in turn leading to cleavage of the inactive form of interleukin- (IL-) 1*β* (pro-IL-1*β*) to its active form (IL-1*β*) [21]. Cytokine production is central to the host inflammatory response during PD and infection [20]. Other caspases involved in inflammation, although less studied and considered involved in the noncanonical inflammasome, are caspase-4, caspase-5, and caspase-11 that are important factors for counteracting Gram-negative bacterial pathogens via induction of cell death and IL-1 release [22, 23].

This review is aimed at illuminating advances in the study of mechanisms of innate and adaptive immune responses after *P. gingivalis* and *F. nucleatum* interactions with the host. We discuss the role of TLRs, the inflammasome, purinergic signaling, cytokines, and chemokines, as well as the innate and adaptive immune cells involved in host resistance to infections by these bacteria. Our review highlights the importance of understanding signaling pathways induced by *P. gingivalis* and *F. nucleatum* that could potentially serve as effective strategies for treating patients with PD.

## 2. *Porphyromonas gingivalis* and *Fusobacterium nucleatum*: Periodontopathogenic Pathogens

*P. gingivalis* is a well-adapted colonizing opportunistic pathogen with the ability to invade gingival epithelial cells [24], periodontal ligament fibroblasts [1], osteoblasts [25], and immune cells [26]. It requires anaerobic conditions for growth *in vitro*, as well as hemin and vitamin K in its nutrient medium. It appears as black-pigmented colonies in blood agar medium attributed to agglomeration of heme groups on its cell membranes [27, 28]. *P. gingivalis* obtains energy through the fermentation of amino acids, thereby allowing survival in periodontal pockets, where there are low sugar levels [11]. *P. gingivalis* is considered an “inflammo-philic” bacterium (from the Greek suffix -philic meaning “attracted to” or “loving”) [29]; it is thought that infections with this organism induce the production of proinflammatory cytokines that damage the host tissue, promoting bacterial survival [30, 31]. Therefore, the conditions of the inflamed tissue favors the nutritional needs of the dysbiotic community, caused by the release of products resulting from tissue destruction, including peptides and components containing heme groups [1, 19, 32]. *P. gingivalis* is normally found in 10%–25% of healthy subjects and 79%–90% of subjects with periodontitis [33, 34]. There is a positive correlation between the depth of the periodontal pocket and the presence of *P. gingivalis* [11].

*P. gingivalis* is considered the keystone pathogen of PD because of its ability to modify the normal oral microbiota composition to one with greater pathogenicity that intensively accelerates bone loss [3, 35]. This periodontopathogen is considered a master of immune system subversion that exploits several sabotage tactics allowing it to evade, weaken, or deceive the host's immune system [36]. *P. gingivalis* possesses several virulence factors, including proteolytic enzymes (e.g., gingipains), capsule, lipopolysaccharide (LPS), fimbriae, nucleoside diphosphate kinase (NDK), ceramide, and outer membrane vesicles (OMVs) [37] (summarized in Table 1).

In a classic study, Socransky et al. analyzed over 13,000 periodontitis subgingival dental plaque samples and grouped the species into bacterial “complexes,” according to the relationship between the different species. The “red complex” is constituted by *P. gingivalis*, *Treponema denticola*, and *Tannerella forsythia*, presenting greater pathogenic potential, and being related to clinical measures of periodontal disease such as pocket depth and bleeding on probing. *F. nucleatum* is part of the “orange complex,” which is a core group that supports colonization of the “red complex” bacteria and is important for the progression of PD [38].

*F. nucleatum* is a human pathogen that is filamentous, Gram-negative, non-spore-forming, nonmotile, and anaerobic [13]. It is a heterogeneous species that belongs to the family *Fusobacteriaceae* and includes five proposed subspecies (*ss*): *ss animalis*, *ss fusiforme*, *ss nucleatum*, *ss polymorphum*, and *ss vincentii* [13, 39–41]. Despite the fact that *F. nucleatum* has been found in various tissues, the most common anatomical site in humans is the oral cavity [13]. As for *P. gingivalis*, this bacterium also presents virulence factors that make it a potential opportunistic pathogen in periodontal infections. *F. nucleatum* possesses several virulence factors, including adhesins (facilitating adhesion and invasion to various cell types, leading to colonization, dissemination, and triggering host immune responses) [13], endotoxins (e.g., LPS) [42, 43], and secretion of serine proteases (responsible for suppressing the nutritional needs of other oral microorganisms) (summarized in Table 2) [44].

There is evidence that synergistic and antagonistic interactions among various microorganisms influence the pathogenesis of PD [45, 46]. *P. gingivalis* suppresses apoptosis in gingival epithelial cells by activating the phosphatidylinositol-3-kinase (PI3K) signaling pathway, thereby favoring its own survival as well as intracellular survival of *F. nucleatum*, as observed in a coinfection subcutaneous chamber model (*F. nucleatum* and *P. gingivalis*) [29]. The interaction between these bacterial species may influence the mechanism of invasion and adhesion of these and other periodontal bacteria in human gingival epithelial cells [2]. *F. nucleatum* was shown to be capable of increasing the invasion of *P. gingivalis* in gingival epithelial cells [2, 47, 48]. In an animal model study, coinfection with *P. gingivalis* and *F. nucleatum* synergistically increased bone loss and exacerbated inflammatory responses when compared to that of monoinfection in rat periodontal tissues [49]. The consequences of these interactions may be caused not only by local inflammation but also by systemic manifestations.

In order to carry out studies with periodontopathogenic bacteria, several bacterial strains are used. The use of bacterial strains from a given type culture is more feasible due to easy access to commercially available sources, as opposed to the more difficult collection of bacteria from clinical subjects. It is important to appreciate that strains of the same bacterial species may have varying characteristics, and therefore, the results generated by one strain are not always transferable to another. It is advisable to use clinical microorganisms as well as those from collections (type cultures) to conduct these studies, in order to compare them with their standards. Notably, it was observed that there were no differences in LPS characteristics and cellular activation when using clinical samples from patients with periodontitis and ATCC strains [50].

*P. gingivalis* strains show genetic variations, and studies have associated these variations with virulence potential, in which certain strains present a greater virulence whereas other strains of the same species display more commensal behavior [51]. Examples of virulent strains of *P. gingivalis* are W83, W50, ATCC 49417, and A7A1, while 381, 33277, and 23A4 are characterized as less virulent strains [52–54]. Regarding *F. nucleatum*, it was shown that all strains significantly increased the phagocytic capacity of neutrophils as well as IL-8 and TNF-*α* production [55]. Interestingly, phagocytosis of *F. nucleatum ss polymorphum* was significantly greater than that of *F. nucleatumssvincentii* and *ss nucleatum* [55]. These studies highlight the importance of choosing the correct strain to perform *in vitro* and *in vivo* studies that mimic the bacterial effects on human oral diseases.

## 3. Immunological Mechanisms Triggered by *P. gingivalis* and *F. nucleatum*

Here, we reviewed pathways used by the host to control *P. gingivalis* and *F. nucleatum* infection as well as how these bacteria subvert these innate and adaptive immune responses.

### 3.1. Pattern Recognition Receptors (TLR2 and TLR4 pathways)

Bacterial components induce innate immune responses through host recognition to pathogen-associated molecular patterns (PAMPs), which are evolutionarily conserved molecules shared by microorganisms, but which are absent in the host. These PAMPs alert the innate immune system to the presence of pathogens. In the same context, in a situation of tissue homeostasis alteration due to microbial invasion, necrosis, cell injury, or stress, the release of intracellular damage-associated molecular patterns (DAMPs) occurs. DAMPs are considered danger signals that alarm the innate immune system and are therefore alternatively called “alarmins” [85].

PAMPs and DAMPs are identified by a wide variety of pattern recognition receptors (PRRs), present in the plasma membrane, cytoplasm, or vesicles (such as endosomes) in inflammatory cells, as well as in resident cells. The recognition of PAMPs and DAMPs by the host results in the induction of signaling pathways such as activator protein 1 (AP-1) and factor nuclear kappa B (NF-*κ*B), leading to the expression of proinflammatory cytokines [86]. The family of PRRs includes Toll-like receptors (TLRs), C-type lectin receptors (CLRs), RIG-I-like receptors (retinoic acid-inducible gene-I-like receptors, or (RLRs)), and nucleotide oligomerization domain- (NOD-) like receptors (NLRs) [86].

TLRs are expressed in oral epithelial tissue, can be stimulated by commensal microorganisms, and also serve to protect the host against microbial infections [9]. The TLR signaling pathway involves the recruitment of the adapter protein containing the TIR (Toll-IL-1 receptor) domain and myeloid differentiation primary response 88 (MyD88) to the cytoplasmic region of TLR, with subsequent activation of NF-*κ*B and induction of proinflammatory cytokines and host defense genes [87].

**Figure 1:**
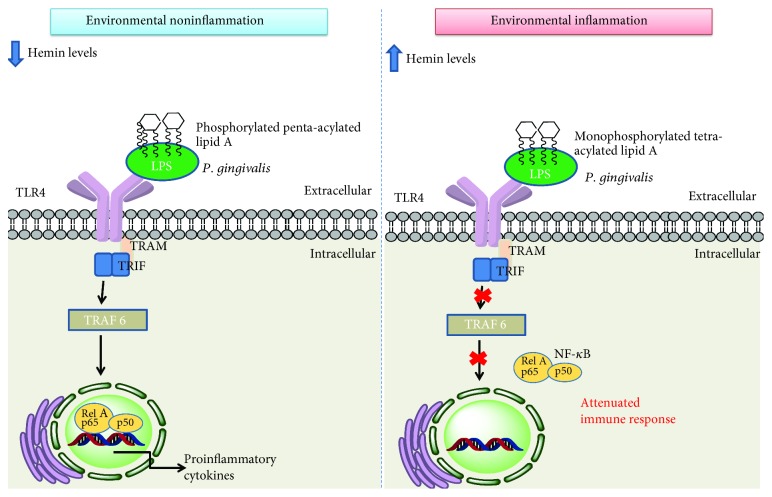
Two forms of *P. gingivalis* lipid A depending on the microenvironment and their interference in TLR4 receptor signaling downstream activation. Legend: LPS: lipopolysaccharide; p65: nuclear factor NF-*κ*B protein p65 subunit; p50: nuclear factor NF-*κ*B protein p50 subunit; Rel A: v-rel reticuloendotheliosis viral oncogene homolog A; TLR4: Toll-like receptor-4; TRAF 6: tumor necrosis factor receptor-associated factor 6; TRIF: TIR-domain-containing adapter-inducing interferon-*β*; TRAM: TRIF-related adaptor molecule.

The interaction of LPS with TLRs is one of the mechanisms of manipulation of the host response used by *P. gingivalis* to facilitate its adaptation and survival [88]. LPS is composed of an antigen O of variable length, a polysaccharide core, and a lipid A moiety. Lipid A is the effector LPS portion that binds to TLR4/MD2/CD14 (TLR4 signaling requires additional costimulation by cluster of differentiation 14 (CD14) and myeloid differentiation protein 2 (MD-2)). The molecular structure of LPS varies depending on the bacterial species [89]. The level of acylation of PAMPs allows the host to discriminate commensal from pathogenic bacteria [90]. *P. gingivalis* LPS differentiates from LPS of other bacterial species through modifications in the O-antigen structure [91–93], as well as modifications in the acylation patterns and in the receptor-activating capacities of the lipid A component [11]. Lipid A from *P. gingivalis* has a penta-acylated phosphorylated structure that activates TLR4 and a tetra-acylated monophosphorylated structure that antagonizes TLR4, thereby attenuating host immune responses. These differences of lipid A structures depend on the microenvironment and hemin concentrations [94]. At high hemin concentrations, conferring high degrees of inflammation, several tetra- and penta-acylated lipid A structures were observed in *P. gingivalis* as opposed to one major penta-acylated lipid A structure at low hemin levels [94] (see Figure 1). Another type of LPS was also identified in *P. gingivali*s, A-LPS, with an anionic polysaccharide linked to lipid A, responsible for serum resistance and cellular integrity; however, it was a weak inducer of cytokine release by human monocytes [91]. The literature remains conflicted regarding signaling of *P. gingivalis* LPS binding to TLR2/4 receptors; this conflict concerns the distinct portions of *P. gingivalis* lipid A that exhibit various receptor binding attributes [88].

**Figure 2:**
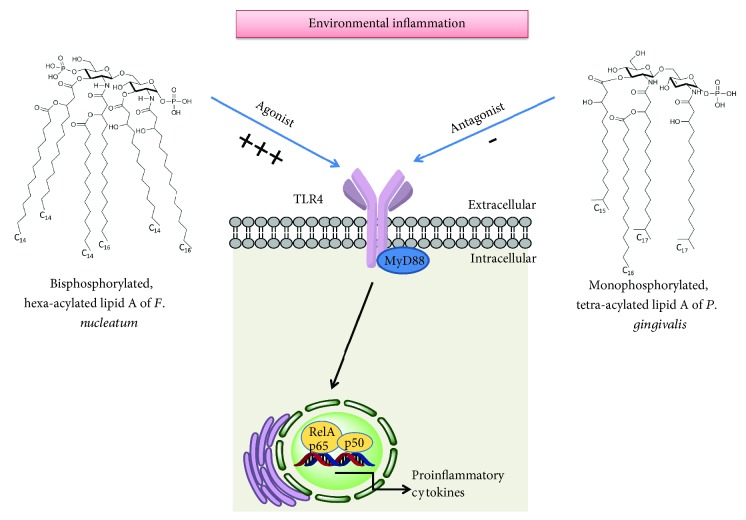
Structural chemical differences in lipid A of *F. nucleatum* and *P. gingivalis* and their interaction with TLR4. Legend: MyD88: myeloid differentiation primary response 88. TLR4: Toll-like receptor-4; -: antagonize TLR4 activation; **+++**: strong TLR4 agonistic response.

*F. nucleatum* lipid A is a hexa-acylated fatty acid composed of tetradecanoate (C14) and hexadecanoate (C16) and is structurally similar to *Escherichia coli* lipid A [95]. This structural similarity may explain the fact that *F. nucleatum* has a strong activity via TLR4 [95] (see Figure 2). These structural differences in bacterial LPS composition may explain why *F. nucleatum* LPS stimulates IL-1*β* secretion more strongly than does *P. gingivalis* LPS [96].

The observations cited above explain why TLR2 predominates over TLR4 in terms of recognition by *P. gingivalis* [36]. Activation of TLR2 by *P. gingivalis* LPS induces two distinct signaling pathways, one of which leads to the synthesis of proinflammatory cytokines, and antimicrobial responses and represents the pathway of CXC chemokine receptor 4 (CXCR4) modulated by *P. gingivalis*. The other cascade involves the proadhesive capacity and pathway crosstalk between TLR2 and the complement system [36]. It has been demonstrated *in vivo* and *in vitro* in neutrophils that *P. gingivalis* inhibits the TLR2/MyD88 signaling which is considered a host-protective pathway, thus avoiding the death of neutrophils infected with *P. gingivalis*. This inhibition occurs through ubiquitination and degradation of MyD88 via E3 ubiquitin ligase Smurf1 dependent of the crosstalk between TLR2 and the complement receptor C5aR (C5aR/TLR2). On the other hand, *P. gingivalis* activates the TLR2/Mal/PI3K signaling pathway that blocks phagocytosis and stimulates inflammation in neutrophils [29, 97]. Because of the effects of *P. gingivalis* gingipains during infection with this pathogen, there are high levels of C5a [97, 98] that could induce alteration of the PI3K pathway leading to inhibition of phagocytosis. The C5aR/TLR2 crosstalk generated by *P. gingivalis* inhibits the activation of RhoA (Ras homolog gene family, member A), cytoskeleton reorganization, and actin polymerization, all of which contribute to phagocytosis. All these effects result in a protective effect, not only for *P. gingivalis* but also for other bacteria of the oral cavity. These modulations by *P. gingivalis* contribute to the persistence of periodontal dysbiosis and the chronicity of inflammation in PD [29].

The crosstalk between the TLR2 and the CXCR4 in lipid rafts of macrophages is another form of subversion of the immune response by *P. gingivalis*, allowing their survival *in vivo* and *in vitro.* The fimbriae of *P. gingivalis* bind to CXCR4 and activate the cAMP-dependent protein kinase A pathway, inhibiting NF-*κ*B and nitric oxide (NO) synthesis [67]. For this reason, fimbriae from *P. gingivalis* are considered molecules with antimicrobial properties [67].

*P. gingivalis* induces the release of IL-1, IL-6, IL-8, and TNF-*α*, promoting inflammatory responses via TLR4/TLR2 in host cells [10, 99–101]. We demonstrated that the induction of IL-1*β* mRNA and protein depended on the activation of TLR2 and MyD88 in murine macrophages; the absence of fimbriae did not affect this stimulus (Morandini et al., 2014), although it is known that the fimbriae of *P. gingivalis* activate TLR2, among others, including phosphoceramides and PG1828 lipoprotein [50]. This was observed in the work of Asai et al., in which *P. gingivalis* fimbriae stimulated the expression of IL-8 in gingival epithelial cells via TLR2 [102].

*P. gingivalis* fimbriae induce the production of proinflammatory cytokines, including IL-6 and TNF-*α*, and they mediate the expression of adhesion molecules, including intercellular adhesion molecule 1 (ICAM-1) [103]. The fimbriae exploit TLR2 signaling to interact with complement 3 (CR3), thereby allowing internalization of *P. gingivalis* into macrophages [104]. This virulence factor reduces the production of IL-12 that may inhibit bacterial clearance and lead to an increase of adhesion of CR3-dependent monocytes to vascular endothelium and transendothelial migration [105].

Both TLR2 and TLR4 are associated with bone loss in an animal model of periodontitis induced by *P. gingivalis* [106–108]. The phosphoglycerol dihydroceramide (PGDHC) from *P. gingivalis* also promotes receptor activator of nuclear factor kappa-Β ligand- (RANKL-) mediated osteoclastogenesis, via interaction with Myh9 (nonmuscle myosin II-A) independently of TLR2/4 [72].

During *F. nucleatum* infection in macrophages, both TLR2 and TLR4 recognize this pathogen and are redundant in terms of the production of IL-6 and TNF-*α* [109]. In macrophages, MyD88 is required for cytokine secretion induced by *F. nucleatum* infection [109]. Furthermore, the same study showed that TLR2/TLR4 and MyD88 were required for the optimal activation of NF-*κ*B and mitogen-activated protein kinases (MAPKs, including p38, extracellular signal-regulated kinase (ERK), and Jun N-terminal protein kinase (JNK)) in response to *F. nucleatum* [109]. Interestingly, using HEK293T cells, which lack endogenous TLRs, it was demonstrated that *F. nucleatum* induced IL-8 secretion [110]. In that study, *F. nucleatum* invasion in the host cell was needed to induce IL-8 secretion, as was p38 MAPK signaling, but not NF-*κ*B or NOD-1 [110]. On the other hand, TLR2-silenced Ca9-22 cells infected with *F. nucleatum* resulted in the absence of IL-6 and IL-8 responses, whereas induction of IL-1*β* remained, suggesting that this bacterium modulates the expression of these cytokines via TLR2 [90].

Antimicrobial peptides, including human beta-defensins (HBD), assist the barrier function of gingival tissue [111]. *F. nucleatum* increased the expression of the HBD-2 and HBD-3, and it is believed that this induction occurs via TLR2, considering that there was suppression of *F. nucleatum*-induced HBD when using TLR2 knockdown cells [112]. Furthermore, FomA, a porin protein from *F. nucleatum*, was shown to induce cytokine secretion through TLR2 signaling [112].

**Figure 3:**
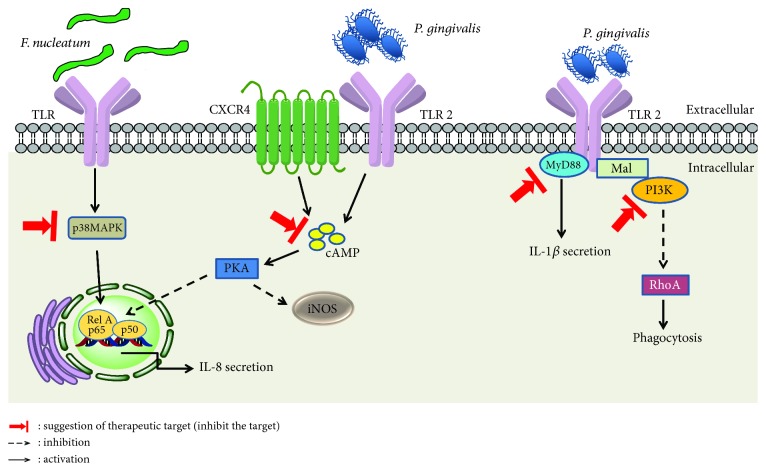
Immune response pathways triggered by the activation of TLR2/TLR4 by *F. nucleatum* and *P. gingivalis* bacteria and possible therapeutic targets. Legend: CXCR4: C-X-C chemokine receptor type 4; cAMP: cyclic adenosine monophosphate; iNOS: inducible nitric oxide synthase; Mal: MyD88 adapter-like; p38MAPK: mitogen-activated protein kinase p38; PKA: protein kinase A; PI3K: phosphoinositide-3-kinase; RhoA: Ras homolog gene family, member A.

Therefore, it is important to understand the TLR2 and TLR4 downstream signaling pathways induced by oral bacteria in cells of the oral cavity and in animal models of PD, because they can potentially be targets of pharmacological treatments. Therapeutic drugs could be designed to target the inhibition of the signaling pathways used by *P. gingivalis* and *F. nucleatum* to survive, as suggested in Figure 3.

### 3.2. Inflammasomes

NLRs (nucleotide-binding oligomerization domain-like receptors) are part of the family of PRRs and can be a part of “inflammasomes” [86, 113, 114]. Inflammasomes are multiprotein complexes assembled in the host cell in response to infection and/or cellular stress that can ultimately lead to a type of cell death called “pyroptosis” and/or proinflammatory cytokine maturation and secretion [19, 115]. Canonical inflammasomes activate procaspase-1 into the mature form caspase-1 while noncanonical inflammasomes involve caspase-11 (in mice) or caspase-4/5 (in humans) [21]. Canonical inflammasomes can be activated by various ligands, and they are named according to the receptor involved in the stress recognition: NLPR3, NLRP1, NLRC4, and AIM2. By contrast, noncanonical inflammasomes are only activated by cytosolic LPS [21]. In the case of the NLRP3 and AIM2 inflammasomes, a PYD-CARD adaptor protein ASC (apoptosis-associated protein with caspase recruitment domain) is required for the assembly and stabilization of these inflammasomes, as reviewed elsewhere [21, 115].

NLRP3 is currently the best characterized inflammasome and is associated with various chronic inflammatory diseases, including type II diabetes, obesity, and intestinal diseases [116]. It is well accepted that two signals are needed to activate the NLRP3 inflammasome: (1) the recognition of a PAMP via PRRs such as TLRs induces NF-*κ*B activation and subsequent transcription of genes encoding NLRP3 and inactive forms of the proinflammatory cytokines, including pro-IL-1*β* and pro-IL-18 [21, 115]; and (2) crystals/particles (such as uric acid and silica), *β*-amyloid, bacteria, viruses, fungi, protozoa, pore-forming toxins, and DAMPs such as adenosine triphosphate (ATP) [23, 115, 117] are recognized by the host cell and activate the inflammasome [115]. Activation of the NLRP3 inflammasome results in the activation of caspase-1 that is responsible for cleaving pro-IL-1*β* and pro-IL-18 to their biologically active forms, IL-1*β* and IL-18 and/or for inducing pyroptosis (to be discussed later in this review [115]). Recent studies showed that activated caspase-1 cleaves gasdermin D, inducing pore formation in the plasma membrane and leading to IL-1*β*/IL-18 release and/or pyroptosis [118, 119].

Studies have examined inflammasome components and their byproducts during periodontitis and infection of cells with periodontopathogens. Human monocytic cells (Mono-Mac-6 cells) infected with *P. gingivalis* showed increased levels of NLRP3 and IL-1*β*/IL-18 but decreased levels of ASC [120]. In THP-1 cells, *P. gingivalis* activated the NLRP3 inflammasome through the TLR2 and TLR4 pathways [121]. This bacterium may downregulate ASC as a mechanism of survival, because ASC is involved in cell death and consequent clearance of intracellular bacteria [122].

We and others demonstrated that *P. gingivalis* infection induced intracellular pro-IL-1*β* production but not IL-1*β* secretion in human gingival epithelial cells [123] and murine macrophages [20, 96, 124]. In fact, in human gingival epithelial cells and murine macrophages, we and others showed that purinergic P2X7 receptor activation by extracellular ATP is necessary for IL-1*β* release after *P. gingivalis* infection [20, 96, 123, 124]. Furthermore, we found that the intracellular processing of pro-IL-1*β* was NLRP3-dependent in murine macrophages [124]. Alternatively, some studies have shown that caspase-8 can also be involved in NLRP3 inflammasome activation and cleaved pro-IL-1*β* in response to TLR4 activation [125, 126].

Furthermore, we showed *P. gingivalis* infection *in vivo* induced IL-1*β* production in order to restrain bacterial infection in a manner that was dependent on caspase-1/11, P2X7 receptor, and autocrine IL-1 receptor signaling [20]. Interestingly, it was showed that *P. gingivalis* mediated inflammasome repression when macrophages were coinfected with *P. gingivalis* and *F. nucleatum* through a mechanism involving reduced endocytosis [96]. These studies highlight the importance of studying infection models in order to understand the pathogenesis and immune responses during periodontitis.

In fact, the inflammasome is very important in the pathophysiology of periodontitis. It is already known that the NLRP3 inflammasome [18] and secretion of IL-1*β* [68] are crucial for the development of PD, because in the absence of NRLP3 or IL-1*β*, there is no periodontitis induced by *P. gingivalis* in mouse models. IL-1*β* also plays a prominent role in promoting tissue pathology and inflammatory responses in periodontal lesions and stimulating the loss of connective tissue and bone [68]. Furthermore, various studies demonstrated increases in the expression of NLRP3, AIM2, IL-1*β*, and IL-18, but not ASC or NLRP2, in gingival tissue from periodontitis patients when compared to healthy individuals [120, 121, 127].

Unlike *P. gingivalis* infections, in murine macrophages and gingival epithelial cells, *F. nucleatum* infections activate the NLRP3 inflammasome leading to pyroptosis and IL-1*β*/IL-18 secretion even in the absence of extracellular ATP, suggesting that *F. nucleatum* provides both PAMPs and a danger signal [96, 128]. In gingival epithelial cells, *F. nucleatum* leads to NF-*κ*B activation, culminating in enhanced expression of the proinflammatory cytokine IL-8 [129] and IL-1*β* secretion [128]. In these cells, *F. nucleatum* infection was sufficient to induce caspase-1 activation in a NLRP3-dependent manner and the secretion of the danger signals ASC and high-mobility group box 1 protein (HMGB1) [128]. Interestingly, we showed that NLRX1 has a dual effect on *F. nucleatum*-infected gingival epithelial cells by upregulating NLRP3-dependent caspase-1 activation but downregulating NF-*κ*B activation and IL-8 production [129]. These results show that inflammasome activation after *F. nucleatum* infection is a robust but complex regulated process. In oral infection in mice, we showed that *F. nucleatum* induced the expression and secretion of several proinflammatory cytokines, including the inflammasome-dependent IL-1*β* [130]. In agreement with *in vitro* and mouse model studies, the expression of the components of the NLRP3 inflammasome was also shown to be increased in periapical lesions in human subjects with periapical periodontitis [131]. Despite the fact that *F. nucleatum* infection *in vivo* was evaluated in some studies, the effects of this bacterial infection *in vivo* with respect to inflammasome activation and modulation still needs to be investigated, because it may be an important therapeutic target.

Regarding inflammasomes other than the NLRP3, it was demonstrated that NLRP6 was more highly expressed in gingival tissues of patients with periodontitis than in healthy controls [132]. The same study showed that pyroptosis of gingival fibroblasts induced by *P. gingivalis* infection was dependent on the NLRP6 inflammasome and caspase-1 activation. Moreover, *P. gingivalis*-infected gingival fibroblasts showed increased levels of IL-1*β* and IL-18 secretion in an NLRP6 and caspase-1-dependent manner [132]. Furthermore, the expression of the AIM2 inflammasome (that recognizes double-stranded DNA) was also more highly expressed in the gingival tissue of periodontitis patients than in controls, as well as in human macrophages infected with *P. gingivalis* [121]. In human macrophages, *P. gingivalis* induced pyroptosis and IL-1*β* secretion in a caspase-1/AIM2 inflammasome-dependent manner [121]. In agreement with these studies, the AIM2 inflammasome was also found in high levels in periapical lesions and was primarily distributed in inflammatory cells [131]. These data suggest that the AIM2 and NLRP6 inflammasomes are involved in the development of PD, highlighting the need to study these immunological pathways during these diseases and periodontopathogen infections, in order to develop therapeutic targets. Together, these data using cell lines, mouse models, and clinical samples demonstrate that inflammasomes play important roles in the pathophysiology of periodontitis as well as during control of *P. gingivalis* and *F. nucleatum* infections. This supports the concept that inflammasome components and/or its byproducts could be potential therapeutic targets for modulating the development of periodontitis and/or controlling infection by periodontopathogens.

### 3.3. Purinergic Signaling

ATP acts as a signaling molecule in various physiological processes, including synaptic transmission, bone formation and resorption, blood pressure regulation, and inflammation [133–135]. Extracellular nucleotides bind to purinergic receptors [136], and these receptors and their subtypes are found in virtually all cell types in mammals [137]. As mentioned above, extracellular ATP is considered a DAMP that can be released from stressed/damaged, infected, or dying cells via various proposed mechanisms, including pannexin-1 hemichannels, connexin hemichannels, or even through the purinergic P2X7 receptors, with consequent autocrine and paracrine cell signaling [138–142].

Purinergic receptors are classified as P1 and P2 receptors [136]. P1 receptors are G-protein-coupled metabotropic receptors that exclusively recognize extracellular adenosine [143, 144]. Their role during periodontopathogen infections will be discussed later in this review. There are two subfamilies of purinergic P2 receptors: P2X receptors that are ionic channels activated by ATP and P2Y receptors that are G-protein-coupled receptors activated by ATP, ADP, UTP, UDP, and UDP-glucose [143, 145]. Currently, seven P2X receptors (P2X1–P2X7) and eight P2Y receptors (P2Y_1_, P2Y_2_, P2Y_4_, P2Y_6_, P2Y_11_, P2Y_12_, P2Y_13_, and P2Y_14_) are known [136, 145]. Among these receptors, the P2X7 receptor has been shown to regulate inflammatory processes that mediate cell death and elimination of intracellular infectious microorganisms; its expression is regulated by inflammatory cytokines [146]. It has been associated with immune responses and inflammation [139, 147, 148], including immune responses against *P. gingiva*lis, as we reviewed elsewhere [19].

We showed that *P. gingivalis* infection induces higher expression of the P2X7 receptor in infected murine macrophages [124] and in the maxilla of orally infected mice [17]. Corroborating our data in mice, we also demonstrated that P2X7 receptor expression levels were lower in patients after conventional periodontal treatment than prior to treatment [17], suggesting that the P2X7 receptor may play a role during PD and infection. Interestingly, P2X7 receptor distribution in macrophages may be modulated by *P. gingivalis* fimbriae, because we showed that receptor expression had a distinct pattern of focus formation in the absence of *P. gingivalis* fimbriae [124]. These data suggest that *P. gingivalis* infection induces P2X7 receptor expression and that *P. gingivalis* expresses virulence factors that allow this pathogen to modulate receptor distribution in the host cell.

In a model of *P. gingivalis* infection *in vivo*, we showed that the P2X7 receptor was required for IL-1*β* production, leukocyte recruitment to the site of infection, and bacterial clearance [20]. Interestingly, the P2X5 receptor was shown to be required for efficient production of IL-1*β* and osteoclast maturation *in vitro*, and that P2X5 receptor deficiency, but not P2X7 receptor deficiency, led to decreased bone loss in an animal model of PD [149]. These data suggest that the P2X7 receptor might be dispensable for the development of periodontitis but it is required for the induction of immune responses and microbial clearance during periodontogenic bacterial infection in murine models. Together, these results suggest that the P2X5 and P2X7 receptors may be novel therapeutic targets in this oral disease.

The ability of *P. gingivalis* to adapt to the gingival epithelium has not yet been fully understood. Among several virulence factors harbored by *P. gingivalis*, it seems that this pathogen evolved to protect itself against extracellular ATP through its nucleotide-diphosphate-kinase enzyme (NDK). NDK is an important virulence factor of *P. gingivalis* that has been shown to cleave extracellular ATP molecules [150]. After *P. gingivalis* infection in gingival epithelial cells, release of ATP occurs and the P2X7/pannexin 1 receptor is activated by autocrine action of ATP; the NDK from *P. gingivalis* accumulates in the cytoplasm [151]. NDK from *P. gingivalis* is carried along myosin-9 filaments and actin filaments to the host cell periphery. Upon translocation to the extracellular environment through the formation of the P2X7/pannexin 1 channel, NDK hydrolyzes ATP, thereby reducing the activation signal of the P2X7 receptor and its downstream signaling events [151]. During infection in gingival epithelial cells, NDK from *P. gingivalis* inhibited ATP-induced reactive oxygen species (ROS) generation, thereby contributing to bacterial persistence [152]. NDK also decreased ATP-induced IL-1*β* release [153] and inhibited ATP-induced host cell death after infection with *P. gingivalis* [150]. Inhibition of these pathways by *P. gingivalis*, by means of its NDK, contributes to intracellular bacterial survival and persistence [151].

*P. gingivalis* also modulates the transcription of forkhead box protein 1 (FOXO-1) genes, leading to the synthesis of antioxidant enzymes such as superoxide dismutase and catalase, restoring the redox balance and preventing long-term oxidative damage. With the inhibition of the toxic response, *P. gingivalis* can survive, replicate, and translocate through adjacent cells, notwithstanding its ability to adapt to the oxidative stress environment [152, 154]. Intracellular *P. gingivalis* inhibited NADPH oxidase (NOX) 2-ROS, followed by suppression of hypochlorous acid production in gingival epithelial cells; these bacteria were present in ER-rich/LC3-positive autophagic vacuoles, considered a new mechanism of bacterial survival [24].

**Figure 4:**
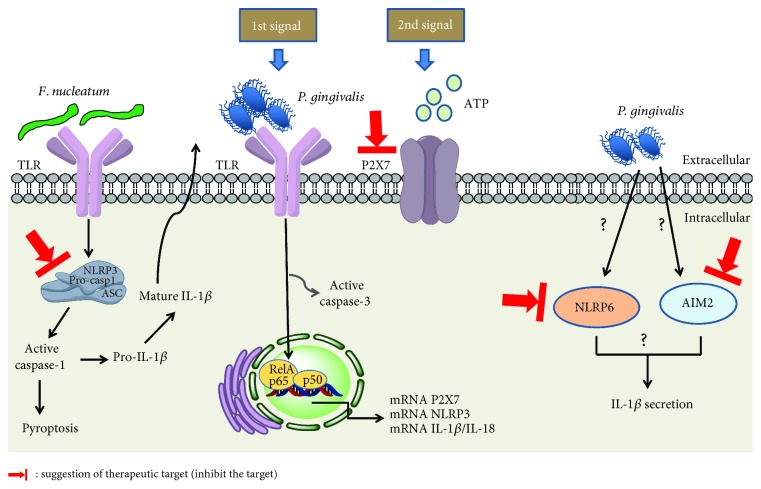
Activation of purinergic P2X7 receptor and inflammasome by the bacterium *F. nucleatum* or *P. gingivalis* and possible therapeutic targets.

P1 receptors recognize extracellular adenosine; they are subdivided into A1, A2a, A2b, and A3, exhibiting varying degrees of sensitivity to adenosine [155]. During gingival epithelial cell infection with *P. gingivalis*, treatment with the specific A2a receptor agonist (CGS-21680) led to bacterial proliferation and increased cAMP levels. However, when a broad-spectrum adenosine agonist (NECA) was used, minimal effects on intracellular *P. gingivalis* levels were observed [156]. These data suggest that adenosine signaling may attenuate inflammatory processes associated with bacterial infection [156], a mechanism of bacterial survival in the buccal mucosa. These results demonstrate the need for further studies aimed at the participation of P1 and P2 receptors in the context of infection by periodontopathogenic bacteria, as well as PD. Furthermore, even though there are no studies of purinergic signaling and *F. nucleatum* to date, the results from our group and others on *P. gingivalis* infection suggest that *F. nucleatum* infection can also be modulated by purinergic signaling infection. In this context, future studies are needed regarding *F. nucleatum* infection and purinergic signaling.  Figure 4 suggests therapeutic targets in the pathways of the activation of purinergic P2X7 receptor and inflammasome by the bacteria *F. nucleatum* and *P. gingivalis*.

### 3.4. Regulation of Caspases Involved in Apoptosis and Pyroptosis by *P. gingivalis* and *F. nucleatum*

Caspases are endoproteases that cleave peptide bonds in a cysteine-dependent and aspartate-directed manner [157]. Caspases can mediate substrate activation and inactivation, and they may also generate active signaling molecules that participate in immune responses, including cell death and inflammation [157]. To date, 11 caspases have been found in humans (caspase-1 to caspase-10 and caspase-14), whereas 10 have been found in mice (caspase-1, 2, 3, 6, 7, 8, 9, 11, 12, and 14) [157]. Caspases are categorized according to their roles in physiology: apoptosis (caspase-3, 6, 7, 8, and 9 in mammals) and inflammation/pyroptosis (caspase-1, 4, and 5 in humans and caspase-1 and 11 in mice).

Caspase-11 has a protective effect during infections in which the bacterium invades the cytosol [158]. This caspase directly binds the hexa-acylated lipid portion of LPS from Gram-negative bacteria, the same component structure that activates TLR4, through its CARD domain [22]. However, species with four acyl groups, although capable of binding, have not been shown to activate caspase-11 [159]. There are some pathogenic bacteria that alter the acylation status of its lipid A, thereby avoiding recognition by cells of the immune system, minimizing the inflammatory process [22]. In this context, it is important to carry out studies that evaluate the possibility of caspase-11 controlling the intracellular proliferation of *P. gingivalis* and *F. nucleatum.*

Apoptosis is a programmed cell death that involves the controlled dismantling of intracellular components while avoiding inflammation and damage to surrounding cells [157]. By contrast, pyroptosis is a nonapoptotic type of cell death that involves plasma membrane rupture and release of proinflammatory intracellular contents [160]. For this reason, pyroptosis usually occurs via noncanonical inflammasome activation by Gram-negative bacteria and is mediated by inflammatory caspase-1 and caspase-4/5 in humans, or by caspase-1 and caspase-11 in mice [118, 119]. Pyroptosis is characterized by the formation of membrane pores, cell edema, and osmotic lysis and release of the cytosolic contents into the extracellular medium [22]. This pathway contributes to intracellular bacterial clearance and destroys any niche formed by intracellular bacterial replication because it causes intracellular bacterial exposure to the extracellular compartment, making bacteria more susceptible to antibodies and attacks by phagocytes such as neutrophils [159]. In this sense, it is advantageous to prevent or delay host cell death in order to preserve their intracellular environment, thereby favoring microbial persistence [19].

Induction or inhibition of apoptosis by bacteria varies according to cell type, bacterial species and strains, duration of infection, and presence of bacterial components (e.g., LPS, proteinases). Cell death by *P. gingivalis* was observed in B cells and human gingival fibroblasts, and inhibition of apoptosis provoked by this bacterium was demonstrated in human monocytes, macrophages, neutrophils, and primary gingival epithelial cells [161–168]. One of the pathways in which *P. gingivalis* prevents the death of gingival epithelial cells either by necrosis or by apoptosis is through the activation of the JAK1/Akt transducer and transcriptional activator 3 (STAT3) pathway, causing upregulation of miR-203 that leads to inhibition of the suppressor of cytokine signaling 3 (SOCS3) negative regulator and subsequent suppression of apoptosis [168–170]. Furthermore, at the mitochondrial membrane, proapoptotic Bad is inhibited and the ratio of Bcl2 : Bax increases, decreasing cytochrome c levels and caspase-3/9 activation, thereby reducing the effect of apoptosis [171].

During infection in murine macrophages, we and others showed that *P. gingivalis* itself did not induce the expression of the activated form of caspase-1, pyroptosis [70], and IL-1*β* [17, 20, 70, 96, 124], requiring a second signal for the activation of these molecules ([17, 20, 96, 124]). However, OMVs isolated from *P. gingivalis* induced the activation of caspase-1 (including pyroptosis) and IL-1*β* in murine and human macrophages [70], suggesting that live *P. gingivalis* negatively modulates inflammasome and caspase-1 activation in favor of its own survival. By contrast, studies using PMA-primed THP-1 cells demonstrated that *P. gingivalis* infection per se induced the activation of the NLRP3 and AIM2 inflammasomes, promoting caspase-1 [121] and caspase-4 activation, leading to pyroptosis in a MOI-dependent manner [172]. Interestingly, high levels of *P. gingivalis* infection did not induce caspase-1 activation or cell death due to a mechanism believed to involve gingipains [70, 173]. Therefore, *P. gingivalis* can modulate activation of caspases involved in apoptosis and pyroptosis depending on the cell line and model of infection. These data agree with those of a study showing enhanced levels of NLRP3 and pyroptosis along with the active forms of caspase-1 and IL-1*β* in the gingival stroma of periodontitis specimens compared to those of healthy samples [174].

*In vitro* studies showed that while *P. gingivalis* can persist intracellularly in macrophages for up to 63 h [175], this bacterium was able to survive intracellularly for up to 8 days in gingival epithelial cells [73]. Indeed, *P. gingivalis* possesses several virulence factors that inhibit host cell death induced by various proapoptotic agents [168, 169]. *P. gingivalis* triggered rapid and reversible surface phosphatidylserine exposure (an apoptosis marker) through a mechanism requiring caspase activation [168]. This opportunistic pathogen can manipulate the host machinery to facilitate its long-term survival by inhibiting the intrinsic apoptotic pathway (cytochrome c release and caspase-3/9 activation) [168, 171]. In fact, it was demonstrated that *P. gingivalis* inhibited chemically induced apoptosis in primary cultures of gingival epithelial cells by blocking the activation of the effector caspase-3 via manipulation of the JAK/STAT pathway that controls intrinsic mitochondrial cell death pathways [169].

Even though *P. gingivalis* does not induce pyroptosis in murine macrophages, *F. nucleatum* infection induces caspase-1 and pyroptosis that is inhibited if *P. gingivalis* is added to the culture [96]. Together, these studies show that *P. gingivalis* is well adapted to survive in various cell types by avoiding the induction of host cell death. These data suggest the need for studies to verify how these bacteria modulate caspases in favor of their own survival, to develop therapeutic targets, and to generate effective treatments for PD.

### 3.5. Adaptive Immunity

The adaptive immune system acts in the context of the chronicity of PD, reinforcing the protection of the host through cellular and noncellular mechanisms [176]. During the development of periodontitis, various T helper subsets predominate in several stages [177]. B cells were observed to predominate in the progression of periodontal lesions, together with Th2 cell profiles [178, 179]. B cells and their antibodies act by preventing bacterial adhesion, by inactivating bacterial toxins, and by acting as opsonins for neutrophil phagocytosis [180].

CD4^+^ T cells are involved directly and indirectly in osteoclastic reabsorption during PD. Various subsets of CD4^+^ T cells promote or suppress immune responses of the host during the progression of periodontitis [181, 182]. Baker et al. demonstrated that animals without MHC-II-restricted CD4^+^ T cells but not MHC-I-restricted CD8^+^ T cells were resistant to oral alveolar bone loss induced by *P. gingivalis* infection, suggesting that CD4^+^ T cells contribute to bone demineralization [183]. Throughout the development of periodontitis, Th1 and Th2 responses characterize disease progression [184], acting as essential immunoregulators and mediators of the initial lesion. Th17 cells may directly or indirectly exacerbate inflammation by modulating Th1 cells or by increasing the synthesis of inflammatory molecules from gingival fibroblasts [185]. By contrast, Treg cells act on the balance of periodontal lesions and become a therapeutic target, allowing the modulation of host immune response, thereby attenuating the tissue damage associated with periodontitis [186]. *P. gingivalis* stimulates increased production of the proinflammatory cytokine IL-17 produced by Th17 cells [187] that act by stimulating the production of inflammatory molecules such as cytokines, chemokines, and other effector compounds, inducing RANKL by osteoblasts, thereby influencing reabsorption of bone [188].

*P. gingivalis* and *F. nucleatum* also induce and evade various adaptive immune responses generated in the infected host. *P. gingivalis* was shown to suppress IFN-*γ*-stimulated release of CXCL9, CXCL10, and CXCL11 from epithelial cells. The inhibition of chemokine expression occurred at the level of gene transcription and was associated with downregulation of interferon regulatory factor 1 (IRF1) and decreased STAT1 expression [189]. In accordance with this idea, *P. gingivalis*-stimulated antigen-presenting cells enhanced Th17 but not Th1 polarization because the bacteria favored the generation of Th17-related cytokines such as IL-1*β*, IL-6, and IL-23, but not the Th1-related IL-12 [190]. Furthermore, when mice were subcutaneously vaccinated with formalin-killed *P. gingivalis* and then were orally challenged with *P. gingivalis*, the vaccination protected the mice from alveolar bone resorption and inflammation. This was due to the downregulation of Th17 immune responses and upregulation of Treg, IL-10, and TGF-*β* production [182]. Regarding the production of the T cell growth factor IL-2, *P. gingivalis* targets its expression at the protein level by inhibiting AP-1 and NF-*κ*B activity, impeding the ability of T cells to sustain stable IL-2 accumulation by means of the bacterial gingipain Rgp [191]. Interestingly, *P. gingivalis* gingipain Kgp was also demonstrated to hydrolyze IgG1 and IgG3 heavy chains *in vitro* [192]. Moreover, cleavage of IgG1 was identified in gingival crevicular fluid from patients with aggressive periodontitis and chronic periodontitis, while no cleavage was detected in healthy controls [193]. In a study involving individuals with untreated and successfully treated chronic periodontitis, both individuals expressed sIgA, IgA, IgG1, and IgG4 against *F. nucleatum* at the same levels but untreated individuals presented sIgA and Th1- (IFN-*γ*- and IgG1-) dominant immune responses [194].

**Figure 5:**
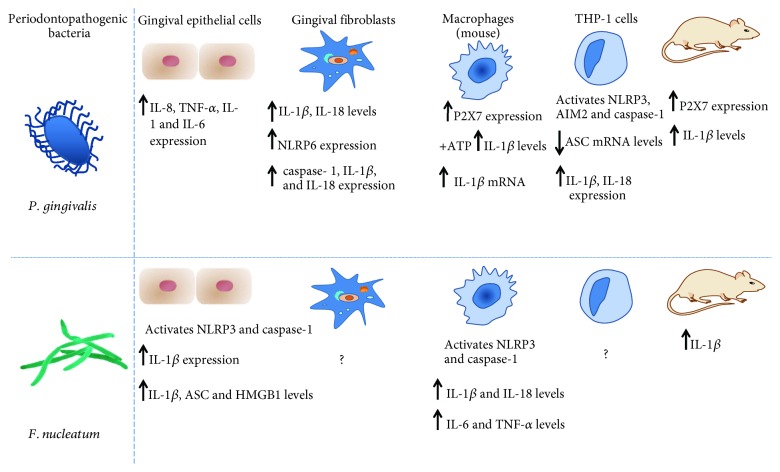
Expression and levels of cytokines, receptors, and inflammasome in immunological and nonimmune cells and in animal models induced by *P. gingivalis* or *F. nucleatum*. Adapted from [195].

Further studies are needed, primarily *in vivo*, to evaluate the role of *P. gingivalis* and *F. nucleatum* in the profiles of immunoinflammatory cells, correlating them with the chronicity of PD such that effective treatments may be developed to prevent the exacerbation of the tissue injury and bone loss.  Figure 5 demonstrates the results discussed in this review.

## 4. Challenges and Perspectives

The role of *P. gingivalis* and *F. nucleatum* in the pathogenesis of PDs is well-documented in the literature; nevertheless, the exact molecular mechanisms induced by these bacteria are not yet fully understood. Studies on TLR2/4 activation by *P. gingivalis* LPS remain to be clarified, because of controversies in the literature. Additional studies in human cells are necessary because the inflammatory potential is higher in these cells than in animal cells. Further studies are also needed to investigate how *F. nucleatum* manipulates downstream TLR2/TLR4 pathways in order to survive and replicate intracellularly. It is known that inflammasomes are involved in the pathogenesis of periodontitis; however, it remains necessary to determine which inflammasomes, in addition to NLRP3, actually contribute to the pathogenesis of PD induced by *P. gingivalis* and *F. nucleatum*. Even though we know that P2X7 receptor is involved in the immune responses against *P. gingivalis*, the role of the purinergic signaling in the context of *F. nucleatum* infection remains unknown. Further research needs to address the role of caspases, especially caspase-11, in the context of inflammation and cell death in *in vivo* and *in vitro* models of PD. More studies that clarify the differences in the immune response of various cells of the oral cavity involved in infections by *P. gingivalis* and *F. nucleatum* are also needed, considering time of infection, MOI, and types of strains to be representative in PD in humans. Because models of coinfections and cocultures are more representative of human periodontitis, it is important that future studies investigate models of *in vivo* coinfection with *P. gingivalis* and *F. nucleatum* in order to better understand the host immune response. Therefore, with more solid literature on these signaling pathways and immune responses during infection with these bacteria, effective treatments for PD may emerge.

## Figures and Tables

**Table 1:** Virulence factors of *Porphyromonas gingivalis.*

Periodontopathogenic pathogen	Virulence factors	Function	Reference
*Porphyromonas gingivalis*	Gingipains	(i) Activates host MMPs; degrades cell-cell components, complement system proteins, cytokines, immunoglobulins, integrins, and collagen; alters cell signal transduction and cellular function (ii) Cleaves T-cell receptors, including CD2, CD4, and CD8 and interferes with the cell-mediated immune response. Cleaves CD14, an endotoxin receptor, resulting in hyporresponsiveness to LPS (iii) Stimulates the expression of protease-activated receptors on neutrophils, gingival epithelial cells, gingival fibroblasts, and T cells, releasing proinflammatory cytokines, to increase vascular permeability and to cause bleeding at the periodontal site and influx of polymorphonuclear leukocytes and degrade fibrinogen	[11, 56–61]
	Capsule (CPS or K-antigen)	(i) Various serotypes of CPS differentially stimulate the release of chemokines by macrophages as well as cytokines by dendritic cells (ii) Encapsulated strains of *P. gingivalis* can also generate greater resistance to phagocytosis by polymorphonuclear leukocytes and differential capacity to adhere to the gingival epithelium	[62–64]
	Fimbriae	(i) Favor adherence to saliva proteins, to the extracellular matrix, to eukaryotic cells, and to other bacteria, contributing to the biofilm generation (ii) Type I fimbriae act on the capacity for invasion and colonization. Type II fimbriae shows greater proinflammatory efficiency	[65–67]
	Lipopolysaccharide (LPS)	(i) Stimulates proinflammatory responses and bone resorption	[68, 69]
	Outer membrane vesicles (OMVs)	(i) The purified OMVs of *P. gingivalis* activates the production of proinflammatory cytokines, signaling of inflammasome, and pyroptosis in macrophages	[70]
	Nucleoside diphosphate kinase (NDK)	(i) Responsible for modulating purinergic signaling and inhibiting proapoptotic actions of primary oral epithelial cells	[71]
	Phosphoethanolamine dihydroceramide (PEDHC) and phosphoglycerol dihydroceramide (PGDHC)	(i) Promotes IL-1*β*-mediated release of prostaglandin E2 (PGE2) in primary cultures of gingival fibroblasts; induces apoptosis in chondrocytes and gingival fibroblasts; promotes osteoclastogenesis mediated by receptor activator of nuclear factor kappa-Β ligand (RANKL) via interaction with Myh9 (nonmuscle myosin II-A) independently of Toll-like receptor 2/4 (TLR2/4)	[72]
	Serine phosphatase	(i) Involved in neutrophil subversion by causing dephosphorylation of the serine S536 of the p65 subunit of NF-*κ*B and prevents translocation of NF-*κ*B to the nucleus, consequently inhibiting IL-8 production	[73–75]

Arg-X: arginine-specific; CPS: capsule; IgG: immunoglobulin G; LPS: lipopolysaccharide; Lys-X: lysine-specific; MMPs: metalloproteinases; Myh9: nonmuscle myosin II-A; NDK: nucleoside diphosphate kinase; NF-*κ*B: nuclear factor kappa-light-chain-enhancer of activated B cells; OMVs: outer membrane vesicles; PAMPs: molecular pattern associated with the pathogen; PEDHC: phosphoethanolamine dihydroceramide; PGDHC: phosphoglycerol dihydroceramide; PGE2: prostaglandin E2; p65: also known as Rel A, nuclear factor NF-kappa-B p65 subunit; RANKL: receptor activator of nuclear factor kappa-Β ligand; TLR2/4: Toll-like receptor 2/4.

**Table 2:** Virulence factors of *Fusobacterium nucleatum.*

Periodontopathogenic pathogen	Virulence factors	Function	Reference
*Fusobacterium nucleatum*	Adhesins	FadA is required for binding and invasion of host cells	[76–78]
	LPS	Stimulates inflammation and bone resorption	[79, 80]
	Serine proteases	Induces damage to host tissue and IgA degradation while favoring acquisition of nutrients	[44]
	Production of ammonium and butyrate	Butyrate and ammonium inhibits the proliferation of gingival fibroblasts	[81]
	Outer membrane proteins (Fap2 and RadD)	RadD and Fap2 function as adhesins, binding to a variety of Gram-positive species and *Porphyromonas gingivalis*, respectively. Both of them induces lymphocyte apoptosis	[82–84]

FadA: *Fusobacterium* adhesin A; Fap2: fatty-acid-binding protein.

## Abbreviations

AP-1: Activator protein 1
ATP: Adenosine triphosphate
ASC: Apoptosis-associated protein with caspase recruitment domain
CPS: Capsule
CLRs: C-type lectin receptors
CARD: Caspase recruitment domain
CD14: Cluster of differentiation 14
CR3: Complement 3
CXCL: Chemokine (C-X-C motif)
CXC4: C-X-C chemokine receptor type 4
DAMPs: Patterns associated with damage
ERK: Extracellular signal-regulated kinase
FadA: Fusobacterium adhesin A
Fap2: Fatty-acid-binding protein 2
FomA: Porin protein
FOXO-1: Forkhead box protein O1
GCF: Gingival crevicular fluid
GM-CSF: Granulocyte-macrophage colony-stimulating factor
HBD: Beta-defensins
HMGB1: High-mobility group box 1 protein
ICAM-1: Intercellular adhesion molecule 1
IgG: Immunoglobulin G
IL: Interleukin
IRF1: Interferon regulatory factor 1
JNK: Jun N-terminal protein kinase
LPS: Lipopolysaccharide
LRRs: Leucine-rich repeats
MAPKs: Mitogen-activated protein kinases
MD-2: Myeloid differentiation protein 2
MMPs: Matrix metalloproteinases
MOI: Multiplicity of infection
MyD88: Myeloid differentiation primary response 88
Myh9: Nonmuscle myosin II-A
NDK: Nucleoside diphosphate kinase
NF-kB: Nuclear factor kappa-light-chain-enhancer of activated B cells
NLRs: Nucleotide-binding oligomerization domain-like receptors
NOD: Nucleotide oligomerization domain
NLRBs: NOD-like receptors or baculovirus inhibitor (BIR) repeats
NO: Nitric oxide
OS: Oxidative stress
OMVs: Outer membrane vesicles
PAMPs: Molecular pattern associated with the pathogen
PBMCs: Peripheral blood mononuclear cells
PGDHC: Phosphoglycerol dihydroceramide
PEDHC: Phosphoethanolamine dihydroceramide
PRRs: Pattern recognition receptors
PYD: Pyridine domain
PGE2: Prostaglandin E2
PI3K: Phosphatidylinositol-3-kinase
PD: Periodontal disease
P2X: The ATP-gated P2X receptor cation channel family
P2Y: Family of purinergic G protein-coupled receptors
p65: Also known as Rel A, nuclear factor NF-kappa-B p65 subunit
Rabs: Ras superfamily of monomeric G protein
RANKL: Receptor activator of nuclear factor kappa-Β ligand
Rel A: v-rel reticuloendotheliosis viral oncogene homolog A
RLRs: RIG-I-like receptors (retinoic acid-inducible gene-I-like receptors)
RhoA: Ras homolog gene family, member A
ROS: Reactive oxygen species
STAT3: Signal transducer and activator of transcription 3
SOCS3: Suppressor of cytokine signaling 3
TRAF 6: Tumor necrosis factor receptor-associated factor 6
TRIF: TIR-domain-containing adapter-inducing interferon-*β*
TRAM: TRIF-related adaptor molecule
TIR: Toll/interleukin-1 receptor
TLRs: Toll-like receptors
TNF-*α*: Tumor necrosis factor-*α*.
